# Evaluation of *in vitro* activity of iclaprim in combination with other antimicrobials against pulmonary pathogens: a pilot study

**DOI:** 10.1099/acmi.0.000027

**Published:** 2019-05-20

**Authors:** David B. Huang, Cyntia de Piano, Sophie Magnet

**Affiliations:** 1 Motif BioSciences, New York, USA; 2 Rutgers New Jersey Medical School, Trenton, New Jersey, USA; 3 IHMA, Rte De I’lle-au-Bois, Switzerland

**Keywords:** iclaprim, synergy, pneumonia, *in vitro*

## Abstract

In this pilot study, the *in vitro* antimicrobial activity of iclaprim, a diaminopyrimidine, tested in combination with other antimicrobials against recent and common Gram-positive and Gram-negative respiratory pathogens, was examined by the checkerboard method. The range of minimal inhibitory concentrations (MICs) for iclaprim against all bacteria tested in the study was 0.03 to >128 µg ml^−1^. Iclaprim exhibited synergy with sulfamethoxazole against 11 of the 16 bacterial strains tested, with mean fractional inhibitory concentration index (FICI) values of 0.2–0.5. Synergy with sulfamethoxazole was demonstrated against all Gram-positive bacteria and selected Gram-negative bacteria. Neither synergy nor antagonism was observed for combinations of iclaprim with ampicillin, meropenem, tetracycline, levofloxacin, aztreonam, piperacillin/tazobactam, colistin, cefepime or gentamicin against any of the bacterial strains tested. The significant reduction in the MIC values observed with the combination of iclaprim and sulfamethoxazole demonstrates that this regimen could be effective against common Gram-positive and selected Gram-negative respiratory bacteria.

## Introduction

Iclaprim is a diaminopyrimidine antibiotic that inhibits bacterial dihydrofolate reductase (DHFR), an enzyme that is important in the folate biosynthetic pathway, and is active against emerging drug-resistant pathogens [1, 2]. Iclaprim contains a stereocentre and is a racemate, a 1 : 1 mixture of (*R*)- and (*S*)-enantiomers. Iclaprim exhibits *in vitro* targeted activity against Gram-positive pathogens that cause pneumonia [1, 3]. Iclaprim also demonstrates rapid *in vitro* bactericidal activity in time–kill studies in human plasma [4]. Iclaprim suppresses bacterial exotoxins (alpha haemolysin, Panton–Valentine leukocidin and toxic shock syndrome toxin-1) [5]. Compared to trimethoprim [the only US Food and Drug Administration (FDA)-approved dihydrofolate reductase inhibitor], iclaprim has lower MIC_90s_, can be given without a sulfonamide, overcomes select trimethoprim resistance and does not cause hyperkalaemia. Iclaprim is administered as a fixed dose over a 2 h infusion and does not require dose adjustment in renally impaired or obese patients.

Because of these findings, iclaprim may be well suited for treating patients with pneumonia caused by susceptible and multidrug-resistant pathogens. In this pilot study, we evaluated the *in vitro* activity of iclaprim and its synergistic effects in combination with other antimicrobials against recent and common Gram-positive and Gram-negative respiratory bacteria.

## Methods

### Collection of bacterial isolates

Non-duplicative and non-consecutive clinical isolates commonly associated with pneumonia were selected randomly (i.e. no iclaprim MIC data were available prior to selection of the isolates) from the International Health Management Associates (IHMA) repository. These isolates were confirmed by IHMA Laboratories using the Bruker matrix-assisted laser desorption/ionization time-of-flight mass spectrometry (MALDI-TOF) biotyper for all isolates except *Streptococcus pneumoniae *, which was identified using standard methodologies, including optochin disc and bile solubility tests. Because this *in vitro* study did not involve human subjects, and the clinical isolates were deidentified from patients with lower respiratory tract infections, no ethical approval number from an institutional review board (IRB) was required. The clinical isolates were collected from humans in 2015 or 2016 from Italy (*n*=2), Germany (*n*=1), France (*n*=2), Hungary (*n*=1), Belgium (*n*=1), Croatia (*n*=1) and Greece (*n*=1). The clinical isolates were *S. pneumoniae* (*n*=1), *Haemophilus influenzae* (*n*=1), *Staphylococcus aureus* (*n*=2), *Klebsiella pneumoniae* (*n*=2), *Pseudomonas aeruginosa* (*n*=1) and *Acinetobacter baumannii* (*n*=2) (Table 1). Seven American Type Culture Collection (ATCC) reference strains were also tested (Table 1). *K. pneumoniae* ATCC 1392171 was a carbapenem-resistant enterobacteriaceae (CRE) and harboured the following: SHV-1 *β*-lactamase , TEM-1 *β*-lactamase, CTX-M-15 *β*-lactamase and KPC-3 carbapenemase. Sixteen isolates and strains were examined in this study, which is a similar number to those examined in prior studies of the *in vitro* activity of antibiotics and its synergistic effects with other antimicrobials [4]. The specific isolates were chosen because they were recent clinical isolates from lower respiratory tract samples (i.e. bronchoalveolar lavage, bronchial brushing, endotracheal aspirate, or sputum) from patients with pulmonary infections; the reference strains are the quality control (QC) organisms recommended by the Clinical and Laboratory Standards Institute (CLSI) guidelines.

**Table 1.** *In vitro* activity of iclaprim and other antimicrobials against Gram-positive and Gram-negative bacteria

			MIC (µg ml^−1^)										
Isolate no.	Organism	Resistance phenotype	ICL	SXA	AMP	MEM	TET	LVX	ATM	TZP	CST	FEP	GEN
ATCC 29213	* S. aureus *	*β*-lactamase-positive	0.06	>128	0.5	0.12	0.25	0.25	>128	0.5	128	2	0.5
ATCC 43300	* S. aureus *	Oxacillin-resistant	0.06	>128	16	2	0.12	0.25	>128	16	128	16	32
ATCC 49619	* S. pneumoniae *	Penicillin-intermediate (altered penicillin-binding protein)	0.03	>128	0.06	0.03	0.12	1	16	0.5	>128	0.03	16
ATCC 25922	* E. coli *	*–*	2	>128	4	0.015	1	0.015	0.25	2	1	0.03	0.25
ATCC 35218	* E. coli *	TEM-1	4	>128	>128	0.015	1	0.03	0.06	1	1	0.03	0.25
ATCC 27853	* P. aeruginosa *	AmpC *β*-lactamase -positive	>128	>128	>128	0.5	8	1	4	2	2	1	1
ATCC 49247	* H. influenzae *	*β* *-*lactamase-negative, ampicillin-resistant	0.25	>128	4	0.06	16	0.015	0.5	0.12	0.5	1	0.5
1252178	* S. aureus *, MSSA	–	0.06	128	8	0.12	0.25	8	>128	1	128	2	0.5
1250165	* S. aureus *, MRSA	Oxacillin-resistant	0.06	32	64	2	0.12	0.12	>128	32	128	16	0.5
1262867	* S. pneumoniae *	*–*	0.5	>128	0.03	0.03	0.12	1	64	0.008	>128	0.03	8
1376187	*K. pneumoniae*	–	4	>128	>128	0.015	1	0.06	0.06	2	1	0.03	0.25
1392171	*K. pneumoniae*	SHV-1, TEM-1, CTX-M-15, KPC-3	>128	>128	>128	32	>128	32	>128	>128	1	>128	64
1303411	*H. influenzae*	*β* *-*lactamase-positive	0.25	>128	128	0.015	0.25	0.015	0.06	0.015	0.5	0.06	1
1485124	*A. baumannii*	OXA-23	>128	>128	>128	128	>128	16	64	>128	1	>128	>128
1496231	*A. baumannii*	OXA-23	>128	>128	>128	128	>128	8	128	>128	1	>128	>128
1370787	*P. aeruginosa*	AmpC *β*-lactamase-positive	128	>128	>128	1	32	0.5	32	>128	2	16	2

AMP, ampicillin; ATM, aztreonam; CST, colistin; FEP, cefepime; GEN, gentamicin; ICL, iclaprim; LVX, levofloxacin; MEM, meropenem; MIC, minimal inhibitory concentration; SXA, sulfamethoxazole; TET, tetracycline; TZP, piperacillin/tazobactam.

### Susceptibility testing

Antibacterial susceptibility testing was measured by IHMA Laboratories (Mothey, Switzerland). Susceptibility testing and checkerboard studies were performed by broth microdilution in accordance with the CLSI guidelines M07-A10 [6] and M100 [7] and the standard operating procedures at IHMA Laboratories. Briefly, stock solutions of antimicrobial agents were prepared 2× and 4× the final concentrations in cation-adjusted Mueller–Hinton broth (CA-MHB) by serial dilutions for MIC determination and checkerboard studies, respectively. The test ranges for MIC determination and checkerboard studies were: iclaprim, 0.002–128 µg ml^−1^; sulfamethoxazole, 0.002–512 µg ml^−1^; ampicillin, 0.002–512 µg ml^−1^; meropenem, 0.002–512 µg ml^−1^; tetracycline, 0.002–512 µg ml^−1^; levofloxacin, 0.002–128 µg ml^−1^; aztreonam, 0.002–512 µg ml^−1^; piperacillin/tazobactam, 0.002/4–512/4 µg ml^−1^; colistin, 0.002–512 µg ml^−1^; cefepime, 0.002–512 µg ml^−1^; and gentamicin, 0.002–512 µg ml^−1^. All isolates and strains were tested in triplicate. *Escherichia coli , K. pneumoniae* and *S. aureus* were tested in CA-MHB. *S. pneumoniae* was tested in CA-MHB supplemented with 2.5–5 % lysed horse blood and *H. influenzae* was tested in haemophilus test medium. For both antimicrobial susceptibility testing and checkerboard interaction studies, the combined antibacterial solutions at 2× the final concentrations were diluted twofold with 50 µl of bacterial inoculum, so that each well contained approximately 5×10^5^ colony-forming units (c.f.u.) ml^−1^. Plates were incubated for at 37 °C for 24 h according to CLSI guidelines and read visually. The QC ranges for iclaprim were those approved by the CLSI and published in M100 (2017). There are no published breakpoints for iclaprim, which is typical for antibiotics in development.

The synergy, indifference or antagonism of each combination was determined based upon the fractional inhibitory concentration indices (FICIs) calculated from the checkerboard interaction results [8]. The fractional inhibitory concentration (FIC) was defined as the MIC of an antibacterial in combination divided by the MIC of the agent alone. For each combination there was an FIC for each agent, i.e. FICA and FICB, whereby: FICI=FICA+FICB. Each combination gave at least one FICI (usually more than one) and an average of the FICIs was used to designate synergy, indifference or antagonism: synergy=average FICI≤0.5, indifference=average FICI>0.5 but≤4 and antagonism=average FICI>4.

### Statistical analyses

No formal statistical analyses were conducted. All isolates and strains were tested in triplicate using the CLSI methodology; modal MIC values are reported.

## Results

### *In* *v* *itro* activity of iclaprim and its synergistic effects in combination with other antimicrobials

Table 1 shows the modal MIC values for iclaprim. All MICs for the ATCC reference strains were within the ranges published by the CLSI in M100 (2017). The range of MICs for iclaprim were 0.03−>128 µg ml^−1^, 0.03–0.25 µg ml^−1^, and 4−>128 µg ml^−1^ against Gram-positive isolates, *H. influenzae* and other Gram-negative isolates (* K. pneumoniae *, *P. aeruginosa* and *A. baumannii *), respectively.

The mean FICIs determined by checkerboard experiments are reported in Table 2. Neither synergy nor antagonism was observed for combinations of iclaprim with ampicillin, meropenem, tetracycline, levofloxacin, aztreonam, piperacillin/tazobactam, colistin, cefepime or gentamicin against any of the bacterial strains tested. Iclaprim exhibited synergy with sulfamethoxazole against 11 out of the 16 bacterial strains tested, with mean FICI values of 0.2–0.5. Synergy with sulfamethoxazole was demonstrated against all Gram-positive bacteria and *H. influenzae* strains tested in this study, as well as against several Gram-negative bacteria, including a highly resistant isolate of *A. baumannii *. The interaction between iclaprim and sulfamethoxazole was indifferent against five of the Gram-negative bacterial strains tested.

**Table 2.** Mean FICI values of iclaprim in combination with other antimicrobials

		Mean FICI									
Isolate no.	Organism	ICL/SXA	ICL/AMP	ICL/MEM	ICL/TET	ICL/LVX	ICL/ATM	ICL/TZP	ICL/CST	ICL/FEP	ICL/GEN
ATCC 29213	*S. aureus*	**0.2**	1.1	1.2	1.5	1.2	1.0	0.8	1.1	1.1	1.2
ATCC 43300	*S. aureus*	**0.2**	0.8	0.9	1.7	1.3	0.9	1.1	1.2	0.8	1.1
ATCC 49619	*S. pneumoniae*	**0.3**	1.1	1.1	1.1	1.1	1.5	1.3	0.9	1.3	1.2
ATCC 25922	*E. coli*	**0.3**	1.2	1.0	1.2	1.1	1.3	1.1	1.1	1.0	1.2
ATCC 35218	*E. coli*	1.2	1.2	0.6	1.2	0.8	1.3	1.0	0.9	1.0	0.6
ATCC 27853	*P. aeruginosa*	1.0	1.1	1.2	1.1	1.1	1.3	1.1	1.0	1.2	1.1
ATCC 49247	*H. influenzae*	**0.2**	1.7	1.4	1.6	1.1	1.4	1.2	1.2	1.3	1.1
1252178	* S. aureus *, MSSA	**0.5**	1.0	1.2	1.5	1.0	1.2	1.5	1.1	1.2	1.2
1250165	* S. aureus *, MRSA	**0.3**	0.9	0.8	1.2	1.0	1.2	0.7	1.1	0.8	1.2
1262867	*S. pneumoniae*	**0.4**	1.0	1.1	0.9	1.0	1.8	1.7	0.9	1.9	1.1
1376187	*K. pneumoniae*	**0.5**	0.9	0.8	1.8	1.1	1.0	1.1	1.3	1.4	1.1
1392171	*K. pneumoniae*	1.1	1.1	1.2	1.1	1.2	1.9	1.8	1.2	1.1	2.0
1303411	*H. influenzae*	**0.2**	0.6	0.8	1.0	0.9	1.0	1.0	1.2	1.1	0.9
1485124	*A. baumannii*	**0.3**	1.1	1.2	1.1	1.2	1.1	1.0	0.9	1.0	1.1
1496231	*A. baumannii*	1.1	1.1	1.2	1.1	1.1	1.1	1.1	0.9	0.9	1.1
1370787	*P. aeruginosa*	0.6	0.9	1.0	1.1	1.1	0.8	1.1	1.2	0.9	1.0

*Note*. Bold values indicate synergy.

AMP, ampicillin; ATM, aztreonam; CST, colistin; FEP, cefepime; GEN, gentamicin; ICL, iclaprim; LVX, levofloxacin; MEM, meropenem; SXA, sulfamethoxazole; TET, tetracycline; TZP, piperacillin/tazobactam.

## Discussion

The study presented here provides updated data indicating that iclaprim remains active *in vitro* against recent common respiratory Gram-positive pathogens. Synergy with sulfamethoxazole was demonstrated against all Gram-positive bacteria, *H. influenzae* and selected Gram-negative bacteria. Interestingly, iclaprim–sulfamethoxazole was not synergistic against *E. coli* ATCC 35218 (which had a resistance phenotype with a TEM-1), whereas the combination was synergistic against *E. coli* ATCC 25922. Neither synergy nor antagonism were observed for combinations of iclaprim with ampicillin, meropenem, tetracycline, levofloxacin, aztreonam, piperacillin/tazobactam, colistin, cefepime or gentamicin against any of the bacterial strains tested. These data provide new information by confirming synergy with sulfonamides against more recent strains compared to an older study from 2007 [9], which also showed that iclaprim was synergistic with sulfamethoxazole and sulfadiazine, confirming the mechanism of action of folate synthetic pathway inhibitors. In that study, neither synergy nor antagonism was observed with macrolides, lincosamides, aminoglycosides, quinolones, beta-lactams, trimethoprim, tetracyclines and glycopeptides. However, iclaprim did exhibit indifference in combination with aztreonam against Gram-negatives and metronidazole against anaerobes.

Although this study did not include trimethoprim and/or trimethoprim/sulfamethoxazole for comparison, recent surveillance studies have shown iclaprim *in vitro* activity compared to trimethoprim and trimethoprim/sulfamethoxazole. A global surveillance of *in vitro* activity of DHFR inhibitors against Gram-positive pathogens showed that iclaprim has remained highly active against a collection of >7500 Gram-positive bacterial isolates during an 8-year period spanning 2004 through 2016. Despite selective pressure with trimethoprim, the only currently approved bacterial DHFR inhibitor, iclaprim maintained low MICs for *S. aureus* (MIC_50_/MIC_90_ values of 0.06/0.12 mg ml^−1^) and *β*-haemolytic streptococci (MIC_50_/MIC_90_ values of 0.015/0.25 mg ml^−1^). Iclaprim was 8–32-fold more potent than trimethoprim alone and had similar activity to trimethoprim/sulfamethoxazole [10].

Synergy may aid the utility of iclaprim in treating pneumonia. The utility of the ability of iclaprim to rapidly and extensively penetrate epithelial lining fluid (ELF) and alveolar macrophages (AMs) (up to 20- and 40-fold higher, respectively, than in plasma [11]) was demonstrated in a phase 2 study comparing the clinical cure rates of two iclaprim dosages with vancomycin in the treatment of patients with nosocomial pneumonia suspected or confirmed to be caused by Gram-positive pathogens. This study indicated that iclaprim and vancomycin have comparable clinical cure rates and safety profiles [12]. The cure rates in the intent-to-treat population were 73.9 % (17 of 23), 62.5 % (15 of 24) and 52.2 % (12 of 23) at the post-treatment test-of-cure visit in the iclaprim 0.8 mg kg^−1^ intravenous (i.v.) q.12 h, iclaprim 1.2 mg kg^−1^ i.v. q. 8 h and vancomycin 1 g i.v. q. 12 h groups, respectively (iclaprim q. 12 h versus vancomycin, *P*=0.13; and iclaprim q. 8 h versus vancomycin, *P*=0.47). The death rates within 28 days of the start of treatment were 8.7 % (2 of 23), 12.5 % (3 of 24) and 21.7 % (5 of 23) for the iclaprim q. 12 h, iclaprim q. 8 h, and vancomycin groups, respectively (no statistically significant differences). A phase 3 study comparing the day-28 mortality and clinical cure rates is planned for iclaprim with respect to the treatment of patients with nosocomial pneumonia suspected or confirmed to be caused by Gram-positive pathogens.

The limitations of this pilot study are: (1) the small number of isolates and clinical strains tested and (2) the fact that only a checkerboard method was used. The use of a larger number of strains segregated as MRSA, quinoline-resistant *S. aureus *, vancomycin-intermediate *S. aureus *, vancomycin-resistant *S. aureus *, penicillin-resistant *S. pneumoniae* and fluoroquinolone-resistant *S. pneumoniae *, and the addition of a time–kill methodology to assess or confirm the synergy of iclaprim in combination with other antimicrobials against a larger number of pulmonary pathogens, would strengthen these and previous observations, and are planned.

Collectively, this pilot *in vitro* study and previous clinical studies support the proposition that, unlike iclaprim alone, iclaprim combined with sulfamethoxazole could be a potential treatment for pneumonia caused by susceptible and multidrug-resistant Gram-positive and selected Gram-negative bacteria. 
